# Proceedings of the Organon Society of Boston

**Published:** 1888-03

**Authors:** S. A. Kimball

**Affiliations:** Secretary


*Secretary.*

---

### THE PROCEEDINGS OF THE ORGANON SOCIETY OF BOSTON.

### REGULAR MEETING, JAN. 12TH, 1888.

Dr. Wesselhœft said that before the reading of the *Organon* began he wished, if it were the pleasure of the members, to speak of the prevalent bronchitis and of a very interesting case of puerperal fever to which he had been called in consultation.

In the bronchitis he had found Alumen indicated in a number of cases, characterized by a scraping from the upper sternum to the throat, with a free expectoration of thick, yellow mucus, paroxysmal cough, morning cough with gagging, aching in chest and in the lower part of the back in region of lower ribs.

The catarrhal symptoms which preceded the cough were running at the nose and sneezing, relieved in the open air. Cepa would help but not cure, and the cough would then come on.

A curious thing was the scraping in chest with the profuse expectoration, for scraping coughs were usually dry.

He then reported the following case of puerperal fever with treatment before he was called in consultation. The patient was a primipara, very slight and delicate. Labor was normal, lasting twenty-three hours; child born at eight P. M. Friday, weighed ten pounds. There was a slight rupture of the perineum; stitches were taken and compress of Calendula applied. Patient went to sleep at midnight; slept until morning.

*Saturday.*—Very comfortable; pulse and temperature normal, but only slept two hours at night.

*Sunday.*—At evening patient had great thirst, face hot and pale, feet cold, uterus much enlarged. Temperature, 102.2. Acon.$^{6x}$ was given and gentle friction was made over uterus.

*Monday.*—Six A. M., pulse and temperature normal; abdomen somewhat tympanitic and sore; lochia slight and offensive. A cool compress, wet in very dilute Arnica, was applied to the abdomen with great relief, and a vaginal douche of warm Calendula water brought away a large clot, half-past six P. M.; temperature, 103.4. Bry.$^{30x}$ was then given on the following indications: Quiet, drowsy, very dry mouth, with thirst for large quantities; soreness of epigastrium, impeding respiration, and stitching pains beneath the lower ribs on deep inspiration; abortive hiccoughing respirations; awaking from sleep; tympanitic distention of abdomen.

*Tuesday.*—At four A. M., copious vomiting of a dark, sour fluid, continuing for two hours; especially excited by motion. Morning temperature, 99.6; pulse, 92; condition about the same; lochia slight. A red spot was now observed on abdomen on the right of umbilicus about as large as the top of an ordinary tumbler. No sensitiveness to touch, no itching or burning. Evening temperature, 102.4; pulse, 107; abdomen much distended and sensitive. During evening and night two involuntary stools, brown, liquid, and undigested.

*Wednesday.*—Three A. M., vomiting like that of previous morning returned, lasting two hours. Seven A. M., temperature, 101.5; pulse, 92; red spot on abdomen increased to size of man's palm; it now involved the umbilicus. The Arnica was discontinued from the compress.

Cold drinks now seemed to aggravate the abdominal and gastric symptoms, while warm drinks relieved. On this indication, and with extreme sensitiveness of the umbilicus, Ars.$^{30x}$ was given. Patient being worse in a few hours, Ars. was stopped and Bry. resumed. Evening temperature, 101.8; pulse, 103.

Stools continued, especially evening and night, preceded by rumbling in abdomen, more in right side, and griping pain in rectum. Stool sudden, forcible, involuntary.

*Thursday.*—Temperature, 99.6 ; pulse. 91; red spot on abdomen increasing, extending toward the left ; great distention of abdomen, pointed at umbilicus, flattened toward right hypochondrium ; extreme thirst and dryness of mouth, can hardly speak ; lower lip swollen, dry, and cracked, mouth and tongue sore ; stools more frequent, quite yellow. On account of aggravation from three P. M. to three A. M., and deep red color of face, Bell.$^{30x}$ was given toward evening in water. (All the preceding remedies had been given in water also.) Evening temperature, 102.8 ; pulse, 110.

*Friday.*—One A. M., on account of temperature not declining as usual, the Bell. was stopped and Bry. resumed, and in two hours temperature fell from 101.4 to 100.3 ; at daylight was over 102. General condition worse ; soreness of mouth and tongue extreme ; yellowish, painful lumps on border of tongue size of a split pea, also on inner surface of lower lip ; lips cracked and bleeding ; red spot on abdomen increasing in size. Urine had been drawn by catheter since birth of child ; no desire to pass it. On account of pain in abdomen a hot compress was applied instead of a cold one with relief.

Dr. Wesselhœft was now consulted at his office. He confirmed the choice of Bryonia but advised its disuse for awhile. He called in the afternoon, took off all compresses and local applications, and gave one dose of Bry.$^{cm}$, dry, and allowed a little scraped apple to be given, which the patient craved. From three P. M. there was a great increase in number of stools, averaging about one every half hour until three A. M. Stools copious, bright yellow, of creamy consistency and quite offensive, accompanied by flatus. Patient complained that the discharge "felt like hot water." Only a slight trace of the lochia and that very offensive. Highest temperature, 102.8 ; pulse, 142.

*Saturday.*—Temperature, 101.6 ; pulse, 130. No marked change ; patient weaker ; bowels quiet during day, except frequent passage of offensive flatus. Dr. Wesselhœft called in the afternoon and prescribed Aloes$^{cm}$ in water every three hours on the following indications : Abdomen conically distended and tympanitic umbilicus projecting. Erysipelas covering abdomen to size of two hands ; patient intensely thirsty, craving large quantities, but satisfied with a few swallows. Diarrhœa the same as above, preceded by desire to pass flatus, which was accompanied by stool. *Both stool and flatus felt hot to the patient.* Desire for scraped apple.

The evening and night were marked by the same increase in stools as the preceding night, but now more frequent and copious than before. Stools involuntary, and following each other in rapid succession. Highest temperature, 103; pulse, 140.

*Sunday.*—Lowest temperature, 100.2; pulse, 120. Patient much reduced by the exhausting discharges of the preceding night.

Morning temperature, 100.2; pulse, 130. Dr. Wesselhœft came early and gave China$^{cm}$ in water every three hours on the following indications: Stools very offensive, with flatus painless, copious, *aggravation at night.* Burning thirst, but frequent small quantities sufficed; appearance of great exhaustion from frequent copious discharges.

During the afternoon slight general improvement. In early evening several small stools, *most of them under the patient's control;* none at all after midnight; night much more comfortable; passed water without a catheter, which she had not done since the birth of the child. Evening temperature, 102.4; pulse, 130.

*Monday.*—Decided improvement; lochia returned; somewhat offensive. Morning temperature, 99.6; pulse, 100. Abdomen much less tense and less sensitive; redness on abdomen fading; patient taking two quarts of milk a day and wanting more. Remedy discontinued Monday evening; patient had had eight doses. Evening temperature, 100.8; pulse, 120.

The condition of the patient steadily improved, and by Wednesday the temperature was normal and pulse 107. No stool for eighteen hours, and the last was somewhat formed and under perfect control, as they all had been since Sunday evening. Abdomen distended, but quite soft, and bears deep pressure without pain; tongue is moist and the sore lumps are disappearing. The patient sleeps quietly most of the time, only waking to wonder if it is not time for her milk.

The lochia became intensely offensive, but the patient was improving all the time and did not notice the odor herself. No remedy was given for it, and in a few days it all passed away.

There was an interesting discussion of this case. Dr. Wesselhœft said the Calendula compress was not unhomœopathic if no remedy was given internally, but the Arnica compresses on the abdomen and the vaginal douche were mistakes. In regard to giving a single dose or repeated doses, it would depend on the case and on the remedy given. In severe cases the remedy would be given often. The China was given eight times and then stopped; Aloes was given every three hours for a day, although she got worse; he thought then it might be an aggravation; he would not change a remedy under twenty-four hours after selecting it with care, and Aloes certainly seemed indicated. The case may have needed Bry., Aloes, and China to cure it; at all events, he did not think the Aloes a mistake, but it was a mistake to leave the Bry. and give the other remedies, Ars., Bell., etc.

Dr. Bell said he thought there was as much importance in knowing how to use the remedies as in knowing the materia medica. He often met men who could talk very well about the materia medica, and seemed to know something of the remedies, but when it came to applying that knowledge in practice they would alternate or change the remedy two or three times in the twenty-four hours, so that the indicated remedy would have no chance if it was used. The great thing was, after having selected a remedy with care, to wait for its effect and not to change too quickly.

In regard to the offensive lochia, Dr. Wesselhœft said the odor was so bad that it was almost impossible to remain in the room with the patient, but she lay there perfectly happy, asking for more milk. He certainly would not interfere with the action of the China as long as the patient was improving. The odor, if it persisted, might call for a remedy later, but it would probably go off in a few days, meantime he allowed no injections or disinfectants of any kind to be used.

Dr. Bell said the old school would probably say that the whole thing was due to not using a bi-chloride solution in the first place to effectually clean out the uterus and vagina, but he thought this method of procedure had already caused too many deaths, and had no more effect in preventing puerperal fever than absolute cleanliness would.

The reading of the introduction to the *Organon* was then commenced, beginning at the twenty-fourth page, and continuing to foot-note twenty, page twenty-nine. It being late, there was not much discussion.

In regard to ailments from fury and anger, Dr. Bell related a case of colic where Bell. seemed indicated, but did not relieve. On finding out, however, that it came on soon after the patient had been very angry, Coloc. was given and relieved very quickly, although it was not a typical Coloc. colic.

In regard to Hahnemann's remark on page twenty-eight, that nature often furnished superabundant osseous matter in the repair of fractures, Dr. Bell said that until recently a callus was thought necessary, but now it is found that there is no callus formed if the bones are kept perfectly still.

Adjourned to January 26th.

### Regular Meeting, January 26th.

Dr. Wesselhœft spoke feelingly of the death of Dr. Lippe, which he announced to the members present. He said that Dr. Lippe was a student of his father's and of Dr. Hering. They organized Allentown Academy, the first homœopathic school in the country.

Dr. Lippe practiced first in a small town in Pennsylvania.

About 1846 he went to Philadelphia and assisted Dr. Hering in his practice, which had grown very large, and on account of the death of his partner and brother-in-law, Dr. Hussmann, Dr. Hering needed some one to help him. At this time Dr. Hering brought the first high potencies from Europe. These were Jenichen's potencies, and Dr. Lippe was the first to teach us how to use them. He was thoroughly imbued with the spirit of Homœopathy ; he never compromised, and was one of whom it could be said, " He never deviated from the law." Dr. Wells and Dr. Bayard are about the only ones now left of the pioneers of Homœopathy in America.

The reading of the introduction to the *Organon* was then commenced. At the twenty-first foot-note Dr. Wesselhœft spoke of a case in which old Dr. Warren, of Boston, made an issue in the leg of a lady with some chronic trouble. It afforded relief for some time, and she kept it open by means of peas, not daring to let it heal, for Dr. Warren told her if it did heal she would die. Dr. Warren died, but she kept it open for thirty years. Finally she came under Dr. Wesselhœft's care for an injury, and the issue healed; the old lady forgot all about it and lived for some time after. In regard to Hahnemann's statement that if a symptom is destroyed without being cured it comes back worse than ever, Dr. Wesselhœft said a patient came to him for treatment for headaches. She had been using bromo-caffeine with apparently good results at first, but the headaches gradually became more severe, and the more she used of the bromo-caffeine the worse they were. So she finally stopped its use and was *cured* homœopathically.

In regard to the suppression of external eruptions several interesting cases were related.

Dr. Wesselhœft spoke of a young married woman with facial acne which appeared after the birth of the first child. She could not wait to have it cured, but it must be got rid of at any cost, so she went to an allopath to have it smeared away. Sometimes such things cannot be smeared away for a long time, but in this case it was accomplished in a short period, and the patient's face was perfectly smooth, but in three weeks she began to cough, and in three months she was dead. Of course, the allopath said it was not due to the smearing; he called it "quick consumption."

He also related another case which occurred last year. A lady after the birth of her first child had a few warty growths on her back and arms, high, elevated, red nodules. These disappeared without treatment. Several years later, after the birth of the second child, she was covered from head to feet with soft, elevated papillæ vascular and smooth, most profuse on the legs and thighs. She had been to all the dermatologists in New York, and they had never seen anything like it. She came to Dr. Wesselhœft. He told her it was evidently a warty growth and surely sycotic in its nature. She was perfectly well, and before beginning treatment thought she would see what the dermatologists in Boston had to say about it. Dr. W. warned her of the danger of letting any one put anything on it, and said if it were suppressed it would probably kill her.

She went to a dermatologist, who told her he had seen such things very often, in fact, had had it himself, and she was persuaded to undergo a course of smearing. Green soap and mercurial ointments smeared the whole thing away, but in a few days facial erysipelas came on and she died in a very short time. They found sugar in the urine a short time before death and said she had diabetes mellitus, the sugar being probably caused by the mercurials used. The whole thing, from the time she saw Dr. W. in his office until she was dead, killed by smearing, was only about three weeks.

Dr. Winn spoke of the case of an old lady with paralysis of the left side from cerebral hemorrhage. She gradually improved, and an eruption developed all over her body with much itching. The more she itched the better she got, and she recovered the use of her arm and leg so that she could get about quite well. Because she couldn't sleep very well on account of the itching, her physician, a so-called homœopath, prescribed a zinc wash. The next day the eruption had all disappeared. In the afternoon she had another cerebral hemorrhage and died that night. Think of it! This was done under the guise of Homœopathy!

Dr. Wheeler spoke of a case where the suppression of an eruption on the legs of a young lady caused a tubercular peritonitis, from which she died in a few weeks.

Dr. Bell spoke of how often we found people, even now, taking gentle laxatives right along, small doses of salts, etc., in order to "keep their bowels open," and they will take Jaborandi to produce a sweat. He said that homœopathic people often take Aconite to get up a sweat. People should be told that a sweat is not necessary for the cure of a cold or any disease. Patients often have a profuse perspiration without relief, and they are very often relieved without any perspiration.

Dr. Wheeler spoke of a case where the family had been trying to "sweat" a patient without success, but after the second dose of Phos.[30] there was a profuse perspiration.

In regard to plugging for nose-bleed, foot-note 24, Dr. Wesselhœft spoke of a case of nose-bleed that lasted a week, not a profuse bleeding, but never stopping and worse at times. Crocus at last cured after several remedies had been given without effect. The patient was a delicate woman with a chronic cough, and Dr. W. refused to stop the bleeding by plugging, although entreated to stop it by any means whatever. A short time after another lady on the same street had an active nose-bleed plugged by an allopath and died of apoplexy in a few days. Dr. W.'s patient then made up her mind that worse things might happen than to wait and be cured homœopathically. She would probably have had some serious lung trouble if the nose-bleed had been suppressed.

Dr. Wesselhœft also spoke of a case of paralysis of one and a-half years' standing. The patient, an old lady, had recovered sufficiently to walk about a little but could not talk much. She had a chronic heart difficulty which interfered with her lying down and caused her great discomfort in breathing; finally she became so much troubled with it that she couldn't lie down at all. At this time a nose-bleed set in; it was not very severe and Dr. W. let it bleed. It bled for twelve hours and then stopped. After that she could lie down with no difficulty in breathing, and this relieved condition continued for some time. It should be stated that Dr. W. had given this patient a remedy a day or two before the bleeding came on and looked upon the bleeding as a curative action, as it probably was. *Dr. Wesselhœft* said *it was always better to do nothing than to do wrong.*

Dr. Wheeler said that when he was at the Harvard Medical School Dr. Calvin Ellis gave no remedies to his typhoid patients, but kept them on a milk diet, and had much better success than when he used to keep them full of drugs.

Dr. Wesselhœft said that in a New York hospital, even now, they give each typhoid patient, on admission, a Calomel purge, and the last report in a journal just received gives eight deaths out of thirty-six patients, twenty-two per cent. They try to explain it by saying that more moribund patients had been admitted than usual. There was no doubt about their being moribund after being subjected to such treatment, and this is the acme of present "medical science !"

Dr. Bell said that he believed any acute disease under allopathic treatment was of longer duration than if left alone. An ordinary tonsillitis under the swabbing and gargling regime is usually from ten days to two weeks in recovering; if left alone it recovers in five or six days; if the homœopathic remedy is given the whole thing is over in two, three, or four days.

In cases that have been drugged severely Nux is not always indicated, not after iodides or bromides; Hepar after bromides, Nux more for the effects of purges and cathartics.

Dr. Wesselhœft said the allopaths talk a great deal about the expectant treatment, that it is much better not to give any drugs, but allow plenty of fresh air and light and pay particular attention to diet. None of them do it, however, although they would probably get better results than they do at present.

Dr. Jameson spoke of a case of deafness treated locally. The deafness was entirely relieved, but brain symptoms soon ensued which necessitated the patient's removal to the Somerville Asylum. As the mental difficulty improved the deafness returned, and when he was in a condition to leave the asylum he was as deaf as he was before he was bungled with.

Adjourned to February 9th.

S. A. KIMBALL, *Secretary.*

